# A Quantitative and Qualitative Analysis of the Phonetic and Phonological Development of Children with Cochlear Implants and Its Relationship with Early Literacy

**DOI:** 10.3390/audiolres15040081

**Published:** 2025-07-03

**Authors:** Marinella Majorano, Michela Santangelo, Irene Redondi, Chiara Barachetti, Letizia Guerzoni, Domenico Cuda

**Affiliations:** 1 Department of Human Sciences, University of Verona, 37129 Verona, Italy; marinella.majorano@univr.it (M.M.); ireneredondi@gmail.com (I.R.); chiara.barachetti@univr.it (C.B.); 2 Guglielmo da Saliceto Hospital, 29121 Piacenza, Italy; l.guerzoni@ausl.pc.it (L.G.); d.cuda@ausl.pc.it; 3 Medicine and Surgery Department, University of Parma, 43121 Parma, Italy

**Keywords:** cochlear implant, children, phonology, phonetics, early literacy

## Abstract

**Background/Objectives**: During the transition to primary school, children with cochlear implants (CIs) may show language and early literacy fragilities. This study has three aims. First, it compares the phonetic and phonological skills of preschoolers with CIs and those with normal hearing (NH); second, it investigates the correlation between phonetic/phonological and emergent literacy skills in the two groups; third, it explores the relationship between phonetic/phonological skills and age at implantation in preschoolers with CIs. **Methods**: Sixteen children with CIs (*M*age = 61 months; *SD* = 6.50) and twenty children with NH (*M*age = 64 months; *SD* = 4.30) participated in the study. Phonetic and phonological skills (phonetic inventories and phonological processes) and early literacy skills (phonological awareness and print knowledge) were assessed. Group differences and relationships between the variables of interest were considered in the two groups. **Results**: A qualitative analysis of phonetic and phonological development showed differences between the two groups. There were also significant differences in early literacy skills (e.g., in syllable segmentation). Significant correlations emerged in both groups between phonetic/phonological skills and early literacy, although in different variables. Significant correlations were also found between age at implantation and the phonetic inventory in children with CIs. **Conclusions**: Preschoolers with CIs display more delays in the phonetic and phonological production skills and more emergent literacy fragilities than NH peers. However, print knowledge did not differ significantly between the groups. Early implantation supports the phonetic skills associated with subsequent literacy learning.

## 1. Introduction

Literature has shown that the auditory advantage given by a cochlear implant (CI) produces positive effects on language development [1,2,3,4], although outcomes are variegated: some children seem able to reach the hearing skills of their peers with normal hearing (NH) [5,6], while up to a third of them present persistent delays [7,8,9]. Many variables contribute to the spoken language development of children with CIs; some of the most influential are medical comorbidities [10], social determinants of health such as socioeconomic status, levels of education and the geographical availability of resources [2], the presence of bilateral hearing access [10,11,12], and environment-related factors [13,14,15,16,17].

However, early implantation improves receptive and expressive language outcomes [9,18,19]. Many recent studies have highlighted that children implanted before 12 months of age [20,21,22,23,24] or at least in the first two years [19,20,21,22,23,24,25] gain better language development [26,27].

It is worth noting that many studies have focused especially on grammar and lexical skills, showing that children with CIs may have difficulties developing morpho-syntactic skills [28,29,30,31,32,33,34], narrative abilities [33] and vocabulary [35].

The few studies of phonetic/phonological development that have focused on early phonetic production have shown that although children with CIs are potentially able to develop fine motor control of the articulatory system when there are no specific concomitant diseases [36], they could struggle to establish acoustic-articulatory mapping because of the absence of feedback of their own vocal sounds. Therefore, in children with CIs, babbling usually starts after implantation, later than expected in normal hearing (NH) peers [37,38]. In the case of early implantation, it is about four months after CIs activation [39,40], although these oral productions seem to be poorer than those of children with NH [41,42].

Thus, while early oral production has been investigated, the later phonetic and phonological development of preschoolers with CIs has been less explored in recent years, especially in Italian children. The relationship between language and reading skills has been fully investigated but is limited to some language dimensions, such as vocabulary and oral comprehension. Considering the importance of early phonetic and phonological skills for emergent literacy and school readiness [43,44,45,46,47], the present study considers these aspects in preschool children with CIs, compared with NH peers, in order to investigate possible differences and associations between the two dimensions of development.

### 1.1. Phonetic Development of Children with Cis

Although technology has improved in recent deca des, CI devices might have slight limitations that affect speech perception and speech production [48,49,50,51]. For instance, difficulties in listening to speech in noisy environments or in competition with other talkers may cause problems in recognizing intonation, discriminating similar acoustic sounds such as /m/ and /n/ [52], /d/ with /g/ [53] and /t/ with /k/ [48,54], and perceiving voicing and voiceless contrast [55]. Some studies [56,57,58] have recognized that some phonemes are acquired later and the phonemic inventories of deaf children seem poorer than those of their peers [59,60,61]. In contrast, other studies, especially in cases of early implantation and after the first years of hearing experience, reported similar phonetic development in children with CIs and NH peers [23,58,62]. Some examples of the phonetic inventories in different languages examined in the literature [23,58,59,62,63,64,65,66,67,68,69,70,71] are reported in Table 1.

Overall, with regard to how phonemes are articulated, stops [23,67,68,72] seem to be easier to acquire for children with CIs in various languages investigated (i.e., Portuguese, English, French, Mandarin). Young CI users can find developing affricate sounds difficult [23,56,67,72,73,74]. Fricatives seem to take longer to establish in deaf children than in their hearing peers [56,65,67,72,74,75], as do the palatal /ʎ/ [67] and the occlusive /t/ [56] and /g/ [63], while other plosives seem to begin to be acquired earlier [67,76]. Conversely, the voiceless fricatives /f/, /s/, /ʃ/ appear in the inventories at 36 months post-implantation, permitted by children with CIs having better motor control than their NH peers [65]. Portuguese children with hearing aids have more difficulties than CI users in producing phonemes /s/, /z/, /Ʒ/, /ɲ/, /l/, /ʎ/ [77].

Concerning place of articulation, nasals [23,59,67,76,78], labials [23,68,78] and alveolar plosives [78] seem to be the most accurate, confirming the “coronal preference” hypothesis of previous studies [79]. Children with CIs tend to promote labials and dorsals over coronals [67]. Among sibilant sounds, alveolar ones seem the most challenging [72]. We can assume that the simplest phonemes to reproduce are those with an anterior point of articulation in the mouth, simple motoric features, and visual cues for their production (e.g., /p/ and /b/) [59]. Considering voicing features, however, voiceless consonants are used more than voiced ones [78].

### 1.2. Phonologic Development of Children with CIs

Some studies have also noted the presence of phonological immaturity in children with CIs [67]. For example, they could have difficulties developing phonological organization strategies, showing fragilities in phonological representations [80,81,82] or present phonological organization strategies different from those of peers with NH [80]. Studies show that children with CIs need more time than peers with NH to recognize phonological competitor words (e.g., phonological assonance, lexical neighborhood) [83] or may have difficulties evaluating phonological similarities between words [80]. Because of these difficulties in distinguishing between similar phonemes, children with hearing loss are at risk for developing speech and sound disorders [8,84,85] or language delays [86,87].

Another aspect to be considered is cross-linguistic differences as reported in the review by McLeod and Crowe (2018) [64] on 64 studies and 27 languages. For instance, in the Italian language, vowel sounds are clearly audible, word structure is quite regular (since it is often made by consonant-vowel syllables), and consonant clusters are, in many cases, composed of two consonants [88]. Considering the word-initial consonant, children are more likely to make substitutions than omissions [65]. Stopping [89,90] and cluster reduction [90,91] are among the most common simplification processes in the speech of children with CIs, while other simplification processes, such as affrication, seem to be found to only a small extent in school-aged children [92]. Shamsian et al. [93] showed that consonant production errors in Persian-speaking children with CIs declined significantly after two years of device use. However, simplifications of words seem to persist until four years after implantation, suggesting that although his phonetic inventory is growing, the child needs time to develop appropriate phonological representations [65,67].

Kindergarten children often struggle to coarticulate two adjacent consonants [94] and present cluster reduction simplification processes, one of the most common phonological processes in the speech of both NH and children with CIs [95]. Considering word-initial/s/-stop cluster simplifications, studies suggest that the perception skills, phonological representations of these structures [96], and the type of errors produced [97] do not differ between CI users and NH children, even though fricative sounds are difficult to perceive for children with hearing impairment.

Speech intelligibility in NH children at four years of age is comparable to the adult model, while this does not occur for children with CIs, whose speech intelligibility is reduced [98,99,100], though it improves with age and device use [67,98]. Some authors have shown that the child’s speech intelligibility is significantly impacted not only by the occurrence of phonological processes but also—especially—by what types of processes are displayed. Cluster reduction, stridency deletion (strident phonemes omission or substitution) and stopping are the more common examples of unintelligible speech samples, proving that intelligible and unintelligible children may develop different phonological simplifications when facing phonological difficulties [101]. However, it could be considered that speech rehabilitation and auditory verbal therapy improve expressive language development of young children with CIs [102,103,104,105].

### 1.3. Early Literacy in Children with CIs

The skills that begin to be acquired before entering school, and influence later reading and writing acquisition [106], comprise the ‘emergent literacy’ skills, some of the main ones of which are print knowledge, phonological awareness, and oral language [107]. Phonological awareness consists of a set of skills that determine not only sensitivity to phonological units (words, syllables, phonemes) but also their manipulation [108]. This process is crucial for decoding (reading) and encoding (spelling) processes [109,110,111,112].

Phonological awareness is an area of deficit in deaf and hard of hearing (DHH) children [113,114,115]. Studies have suggested that whereas these children follow the same patterns as NH children in developing this skill, it develops more slowly [106,116] and remains fragile through the preschool years [113].

Tomblin et al. [117] investigated spoken language and phonological processing in DHH children, not only confirming the presence of fragilities in emergent literacy skills but also noticing that these skills are poorer when the degree of deafness is more severe. Phonological awareness typically evolves from larger to smaller units, with increasing degrees of complexity during a child’s growth [115]. Whereas children seem to develop a predisposition to recognizing the syllabic structure of words and developing syllable and rhyme awareness in preschool [118,119], phonemes-based analysis skill develops later, being completely gained by 7 to 8 years of age [119], partially promoted by academic skills [120,121]. Studies by James et al. [116] confirmed that the development of phonological awareness in children with CIs follows the same sequence of syllable, rhyme, and phoneme awareness as that of NH children.

Findings on syllabic skills seem mixed: some (e.g., Lee) [122] show that children with CIs achieve lower scores on syllable elision and blending tasks than their NH peers, independently of age of implantation, while others (e.g., James et al.) [116] found good results in syllables or word analyses.

Phoneme awareness development seems challenging because of the compromised auditory experience in the first years of life [123,124] and because the technological limitations of CIs themselves [23] negatively impact phonemic categorization [108]. For these reasons, phonetic structure awareness is weaker in children with CIs than in hearing peers [108,116,125,126] during preschool, and these fragilities seem to persist over time [127].

Print knowledge is considered an area of strength for children with CIs since they show comparable results with children with NH [115], probably because of the exclusion of the hearing canal in completing these tasks. This skill is important because in learning to recognize written words, the children start making ‘paired-associated learning’, creating a correspondence between visual information (letter shape) and sound information [128], in the first stage of the grapheme–phoneme mapping process.

### 1.4. The Relationship Between Phonetic/Phonological Development and Early Literacy

A relationship between lexical skills and emergent literacy has been suggested by Lund [129], confirming that oral language is directly connected to written language [115].

Antia and colleagues [130] focused on the possible relationships between phonetic/phonological skills and early literacy in deaf and hard-of-hearing spoken-only and speaking-and-signing children; they noticed that the latter group gained lower scores than the former in blending tasks and hypothesized that speech production issues could cause this weakness.

A relationship was found between phonology and reading skills in the hearing-impaired population [131]. NH and CI children both seem to need the same skills for building reading and writing abilities [132].

However, the relationship between phonological processing and literacy in children with CIs is not fully understood [133]. Some authors have argued that phonological awareness is directly correlated with literacy outcomes [133] since the better this skill is, the better the reading results are [134]. Other studies, however, suggest that the relationship between the two is more complex, due mainly to two factors. Firstly, phonological awareness is higher in opaque orthography languages than in transparent ones (such as Italian). Secondly, phoneme-based representations are more related than syllable-based ones to literacy outcomes [135].

In transparent orthographic system languages such as Spanish or Italian, phonemic representation deficits can be partially compensated through reading. If the child’s mental representation of a speech unit (for example, a word) has some imperfections (for example, the difficulty in perceiving a sound), visual representation supported by reading can help her/him to improve its mental representation, leading to better phonemic awareness skills. However, deaf children seem to have phonological representation of sounds and rely on phonetical information (not orthographic one) when completing meta-phonological tasks such as determining the number of phonemes in stimuli [136].

Phonological processing plays a crucial role in reading development in children with NH [137,138], but studies on how unaided deaf children activate phonological representation in the reading process offer differing results. Whereas some studies have stated that the phonological process used in reading is directly correlated with better reading results [139,140], others have observed that deaf readers have less access to phonological processing than peers with NH [141]. Others have suggested that deaf readers could bypass phonological processing, relying on morphemes and orthography in the text in the reading process [142,143], recognizing words or part of a word in their overall visual processing, with greater use of processes described by Uta Frith in the logographic and orthographic phase of reading development [144]. Finally, other studies recognize that these children use the same processes as typical hearing children but struggle more with phonological analysis of the items than with direct access to the orthography of the word [123].

Visual and linguistic processes are strongly correlated in the first stages of literacy. Specifically, when the child approaches reading or recognizing graphemes, the brain areas involved in the visual analysis of the stimuli and spoken language (semantic meaning and articulation) are both activated [145].

To our knowledge, only a few studies in recent years have explored the development of phonological awareness in children with CIs [109]. Another question of interest is whether different language domains are strictly connected during their development. Ingvalson et al. [115] found that some language areas co-develop during childhood; the present study asks if this relationship could also pertain to phonetic/phonological and emergent literacy skills in children with CIs.

### 1.5. The Present Study

The present study assesses the phonetic/phonological skills and early literacy of Italian children with CIs, comparing their performance with those of age-matched NH children. Specifically, the aims are as follows:

1. To compare the phonetic/phonological skills of children with CIs with those of NH peers. According to the literature [59], we expected children with CIs to show delay in phonetic and phonological indices. Specifically, they could have difficulties in mastering more complex sounds and could produce a higher number of phonological processes than peers with NH.
2. To investigate early literacy (phonological awareness and print knowledge) in children with CIs and their NH peers. According to the literature, we expected lower scores for children with CIs in phonological awareness tasks (syllable segmentation and syllable blending), while in print knowledge we could expect the scores to be more similar due to the facilitation of the visual stimuli presented [115]. Children with CIs could rely more on non-acoustic cues (particularly visual analysis of the stimuli) in early literacy tasks.
3. To investigate the relationship between phonetic/phonological and early literacy skills in children with CIs and children with NH. According to the literature [142,143], we could expect that the relationship between these domains could be different between groups. Specifically, the print knowledge of children with CIs could be less related to the phonetic and phonological domain due to the visual facilitation of the task.
4. To investigate the relationship between phonetic/phonological development in children with CIs and individual factors. According to other studies [23,26], children with early implantation could display higher levels of phonetic/phonological development.

The study was approved by the Ethical Committee of the University of Verona (Italy) and the Ethical Committee of the “Guglielmo da Saliceto” Hospital (Prot. N. 1053/2019/OSS/AUSLPC).

## 2. Materials and Methods

### 2.1. Participants

The children participated in a broader project assessing oral language skills in preschoolers with CIs and with NH [17,146]. Two groups of preschoolers participated in this study. In total, 16 children with CIs (hereafter, the CI group) (9 girls; mean chronological age = 61 months, *SD* = 6.90; mean age at implantation = 21.94 months, *SD* = 11.07) with profound sensorineural hearing loss (SNHL) were recruited from “Guglielmo da Saliceto” Hospital in Piacenza, Italy. Nine children had genetic SNHL due to mutations of the connexin 26 gene; the remaining seven had unknown congenital aetiology. Three children wore unilateral CIs, seven wore bilateral CIs, and the remaining six wore a CI and a hearing aid on the unimplanted ear (bimodal stimulation). The children’s mean unaided Pure Tone Average (PTA) [147] in the implanted year was 125.38 dB (*SD* = 6.84). All children underwent speech therapy following an Auditory–Verbal Therapy (AVT) approach [148] after being fitted with hearing aids (i.e., by the first year of life).

The second group of participants (hereafter, the NH group) consisted of 20 age-matched children (11 girls; mean chronological age = 64 months, *SD* = 4.30) with NH, recruited from an infant school near Vicenza in the Veneto region (Italy). The two groups are chronologically homogeneous, as no significant differences in age emerged between them (*U* = 112, *p* > 0.05). All the children are born at term and do not display evidence of neurodevelopmental disorders or other developmental difficulties. Children from both groups are monolingual.

For what concerns the socio-economic characteristics of the two samples, the mean years of education was 12 (*SD* = 3.5) for the mothers of the CI group. According to the Italian educational system, this corresponds approximately to a completed upper secondary school education. For the mothers of the NH group, the mean years of education was 16.8 (*SD* = 2.77), which corresponds approximately to the completion of a bachelor’s degree plus some postgraduate education.

### 2.2. Procedure

All the children were individually assessed on phonetic/phonological skills and early literacy. The assessment was administered in two sessions of around 40 min each. Children were administered the phonetic and phonological measures first, then the early literacy measures (CMF test first, then syllable identification and vowel identification tasks).

### 2.3. Instruments

#### Phonetic and Phonological Measures

The PFLI (Prove per la Valutazione del Linguaggio Infantile) [149] test is an Italian instrument used for the clinical assessment of phonological fragilities in spontaneous language in young children (from 2 to 5 years). In this study, a short form of the instrument was administered following a semi-structured procedure; it consisted of 32 pictures of scenes to be described by the child. For each picture, the administrator asked the child: “Tell me what you see in this picture”, to elicit the production of words containing multiple occurrences of all language phonemes. The procedure was videotaped for each child. Each child’s spontaneous language sample was transcribed following the International Phonetic Alphabet (IPA) [150] and according to the Italian language. The words pronounced, the repetitions, and the unintelligible words were included in the transcription.

Then, we coded the phonemes in the collected samples under three headings: acquired phonemes (phonemes that are present in at least three words), emergent phonemes (phonemes that are present only in one or two words), and absent phonemes (phonemes not present). According to the number of words collected in the sample, the presence/absence of phonemes was classified as follows.

In a sample of less than 100 words, the phoneme was considered acquired if it occurred at least three times in two different positions (initial or median position of a word) in three different words.

In samples of 101–150 words, the phoneme was considered acquired if it occurred at least twice in the initial position of a word and twice in the median position in at least three different words.

In samples of more than 150 words, the phoneme was considered acquired if it occurred at least three times in the initial position of a word and three times in the median position.

Finally, we counted the following:

- The number of correct words, considering just one occurrence for every word;
- The number of simplified words, considering just one occurrence if a single word was simplified multiple times with the same phonological result;
- The number of unintelligible words: words that cannot be recognized after three hearing attempts by two different listeners.

For the phonological descriptive analysis, we considered the phonological simplification processes for every simplified word in the sample collected, excluding simplifications of absent/emergent phonemes. These simplifications were as follows: stopping (a fricative or affricate sound is substituted by a stop consonant), affrication (a non-affricate sound is substituted by an affricate), deaffrication (an affricate sound is substituted by a fricative one), gliding (when a/ʎ/ becomes a/j/), fronting (a velar sound is substituted by an anterior one), backing (an anterior sound is substituted by a velar one), devoicing (a voiced sound is substituted by its voiceless correspondent), voicing (a voiceless sound is substituted by its voiced correspondent), palatalization (a non-palatal consonant becomes palatal), weak syllable deletion (deletion of a non-stressed syllable), consonant or vowel omission, epenthesis (phoneme addition within the word), metathesis (phonemes shift within the word), diphthong reduction (cancelation of a vowel in the diphthong), consonant harmony (assimilation of a consonant sound with another consonant within the word), consonant substitution, cluster reduction (cancelation of a consonant forming a cluster), atypical processes and idiosyncratic processes.

### 2.4. Early Literacy Measures

We considered two dimensions of early literacy: phonological awareness (syllable segmentation and syllable blending) and print knowledge (syllable identification and vowel identification).

We considered only syllables for phonological awareness because, as reported by Liberman et al. [109], phoneme segmentation and blending skills are usually developed later.

### 2.5. Phonological Awareness Task

Subtests of the Metaphonological Skills Evaluation Test (CMF) [151] were used for assessing metaphonological skills. We administered the syllable blending and the syllable segmentation tasks, each comprising 15 words that the child must blend or segment. The two tasks are scored by counting the number of correct answers. Raw scores were considered for this study.

### 2.6. Print Knowledge Tasks

Syllable Identification task. For this task, we chose twenty syllables based on the principles of maximum contrast for sound and form, based on the syllabic method developed by Bertelli et al. [152], i.e., the maximum perceived discrepancy for sound and form of a given syllable, C1V1, C2V2. (for example, in Italian: SI, MO, RE), and on maximum word generativity (i.e., the highest number of words a group of syllables is potentially able to compose) [153]. The syllables are presented in triplets to the child on a screen. The researcher pronounces a syllable, and the child is asked to point to the correct syllable.

Vowel Identification task. In this task, the five vowels in Italian a, e, i, o, and u are presented to the child on a screen in this order, one at a time. The child is asked to tell which vowel is shown on the screen.

These two tasks are scored by assigning 1 point to each correct answer.

### 2.7. Reliability

Cohen’s k was used to examine the inter-observer reliability of the coding of the children’s phonetic inventories. Analyses of reliability for the coding of phonemes were run on a random sample of 20% of raw scores for each of the two groups. For the coding of the children’s acquired/emergent/absent phonemes in their phonetic inventories, the average kappa was found to be strong for children with CIs (*k* = 0.81) and almost perfect (*k* = 0.98) for children with NH [154].

### 2.8. Data Analysis

Nonparametric independent *t*-tests were conducted to show differences between the CI and NH groups in scores of phonetic/phonological skills and early literacy. Two separate correlation matrices for the two groups were computed to observe associations between the children’s phonological skills and measures of early literacy. A correlation matrix was then computed to observe associations between the individual characteristics of children with CIs (i.e., age at implantation, acoustic measures), phonological skills, and measures of early literacy. All statistical tests were run using version 2.3 of Jamovi software [155].

## 3. Results

### 3.1. Descriptive Statistics

**Figure 1:**
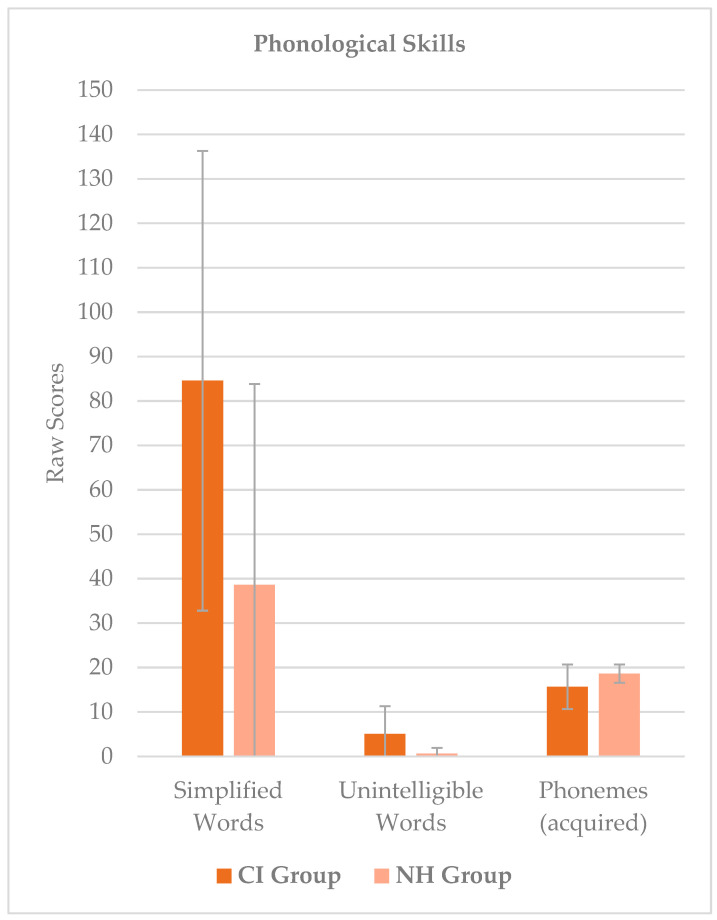
Mean scores and standard deviations split by the two groups for phonological skills as measured at the PFLI test.

**Figure 2:**
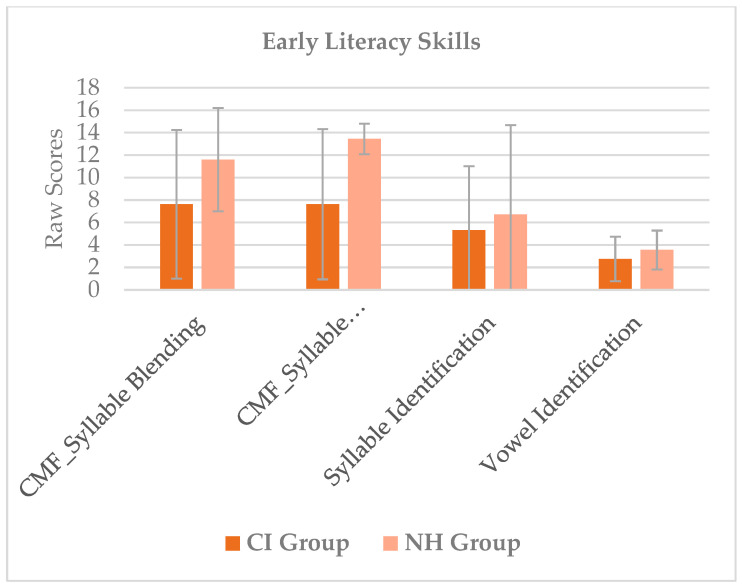
Mean scores and standard deviations split by the two groups for early literacy skills as measured at the CMF test and at the syllable identification and vowel identification tasks.

Figure 1 shows descriptive statistics for both groups considering phonological skills as measured at the PFLI test. Figure 2 shows descriptive statistics for both groups considering early literacy skills as measured at the CMF test and using syllable identification and vowel identification tasks.

Considering the percentage of children using a certain type of simplification process, children with NH present the same number of processes as children with CIs, or fewer, except for palatalization (found in just one child with NH) (see Appendix A).

Regarding systemic processes, both groups present comparable percentages of children using gliding and frication processes, while more children with CIs than children with NH made substitutions between fricative and plosive sounds (stopping) and between fricative and affricates sounds (affrication). While 20% of children with NH used fronting processes, twice as many children with CIs used them; similarly, whereas only 5% of children with NH used backing processes, 27% of children with CIs did so.

Children with CIs made significantly more syllable deletions than peers with NH (respectively, 73% and 15%). A greater difference in making epenthesis occurred between the NH children (20%) and children with CIs (80%). Half of the children with CIs used processes that cannot be specifically analyzed (idiosyncratic processes). The NH and CI groups both presented mostly substitutions and consonant cluster reduction (the two processes comprise almost half of the simplified words). One child’s speech had no phonological processes, since his phonetic inventory and speech production were too poor to consider it for a phonological analysis.

### 3.2. Comparisons Between the CI Group and the NH Group in the Phonological Skills and Early Literacy Skills

First, we considered the differences between the CI and NH groups in phonological and early literacy skills. As observed in Table 2, significant differences emerged between the two groups in their phonological characteristics and in early literacy. In particular, the NH group displayed fewer simplified words than the CI group (*U* = 67.0, *p* = 0.003), fewer unintelligible words (*U* = 80.0, *p* = 0.005), and more acquired phonemes (*U* = 85.0, *p* = 0.016). In addition to that, the NH group displayed higher scores than the CI group in the CMF syllable segmentation task (*U* = 84.5, *p* = 0.015). As regards the CI group, the kind of errors made by the children at the CMF syllable segmentation task were as follows: phonetic and syllabic segmentation mixing and vowel reduplication. We have converted raw scores into percentile ranks, and we found that six of them were below the 5th percentile, one of them was between the 5th and the 10th percentile, three of them were between the 10th and the 25th percentile, and five of them were between the 25th and the 50th percentile. No significant differences were found between the two groups’ scores in the syllable blending task or in the syllable identification and vowel identification tasks (all *p* > 0.05).

### 3.3. Associations Between Phonological Skills and Early Literacy Skills in the CI and NH Groups

Two separate Spearman correlation matrices were computed to explore associations between phonological and early literacy skills in the CI and NH groups. As observed in Table 3, in the CI group, significant moderate negative correlations were found between the number of unintelligible words at the PFLI and scores in the CMF syllable segmentation (*rho* = −0.58, *p* = 0.02) and syllable blending tasks (*rho* = −0.55, *p* = 0.03).

As observed in Table 4, significant moderate correlations were found in the NH group between the number of acquired phonemes in the PFLI and scores in the syllable identification (*rho* = 0.45, *p* = 0.05) and the vowel identification (*rho* = 0.50, *p* = 0.02) tasks. Furthermore, a significant negative correlation emerged between the numbers of simplified words and scores in the vowel identification task (*rho* = −0.48, *p* = 0.02).

### 3.4. Associations Between Age at Implantation, Phonetic/Phonological Skills, and Early Literacy of Children with CIs

A Spearman correlation matrix was computed to explore associations between age at implantation, phonetic/phonological skills, and early literacy in the children with CIs. Significant moderate negative correlations emerged between age at implantation and the number of acquired phonemes produced at the PFLI (*rho* = −0.53, *p* = 0.03) and significant positive correlations between age at implantation and scores in the syllable identification task (*rho* = 0.59, *p* = 0.01).

## 4. Discussion

The present study analyzed the phonetic/phonological skills and emergent literacy in children with CIs and NH. The relationship between these dimensions of development, and the relationship between phonetic/phonological development and age at implantation, were also considered.

Regarding phonetic/phonological development, since literature shows contrasting results about this topic, our study can better clarify both the timing and characteristics of expressive language. On the one hand, our results confirm that, as reported by Sohrabi & Jalilevand [59], although children with CIs build up phonetic inventories, their sound repertoire is poorer than that of their peers with NH. On the number of phonemes mostly acquired (≥70% of stability among children), in our sample, children with CIs show a repertoire of 16 phonemes at 21 months after implantation, while their peers with NH have 19 phonemes. On the other hand, our results contrast with Spencer and Guo [65], who observed that at 36 months after implantation, children with CIs and NH from English-speaking families showed repertoires of 13 and 10 phonemes, respectively. However, in line with their study, we found the presence of the voiceless fricatives /f/, /s/, /ʃ/ in the CI group’s inventories. Notably, whereas children with NH mostly master sounds, we found more heterogeneity among the percentage of children with CIs who had completely acquired the sound, those who had partly acquired it, and those who did not produce it in their speech.

Furthermore, the present study could add new detailed information about phonetic and phonological characteristics in Italian that show similar characteristics with other languages [64]. In our study, CI users seem to mainly acquire simpler modes of sound articulation, such as nasals and stops, in line with the findings of Yang and colleagues [72], while the stabilization percentages of fricative and affricate sounds are heterogeneous, consistent with the difficulties with these categories of sounds reported in the literature [23,72,73,74]. In line with Sohrabi and Jalilevand [59], easy-to-produce and more articulatory visible sounds such as plosives and nasals are acquired equally by both groups, suggesting that these visual cues help children with CIs to master phonemes articulation.

Considering the place of articulation, children with CIs have more difficulties in acquiring those phonemes that present less visible cues (such as velar/g/, which appears later in CIs inventories, according to Iyer et al. [63], and more complex ones. This suggests an immaturity in their phonetic development. Plosives and nasals seem to emerge earlier, as noted by Lynce et al. [67] and Warner-Czyz & Davis [76]. Bilabial phonemes such as /b/, /p/ and /m/ are mainly acquired by children with CIs, who present fragilities in mastering palatal /ʎ/ (acquired by only 12.5% of CI users), in line with Lynce and colleagues [67], /ɲ/ (acquired by 62.5%) and/w/ (acquired by 56.25%), and alveolars /z/ (emergent or absent in all the CI users) and /ʦ/ (acquired by 37.5%). Notably, while the NH children all acquired specific sounds, no child with CIs reached 100% acquisition for any of the phonemes.

Overall, in the NH group, most phonemes have been acquired, while in the CI group there is more variability: a group of sounds is not stably present in their inventory yet, suggesting that their phonetic inventory is still building up. While this is in line with some studies [59,60,61]. It contrasts with the findings of Iyer et al. [63], who stated that all these sounds would be acquired in the first two years of hearing age. Children with NH seem to struggle to master fricative and affricate sounds, rather than plosive and nasal sounds.

As stated in the introduction, the most challenging sounds for children with CIs seem to be the fricatives. While children with NH have all acquired or at least present the fricative/z/, none of the children with CIs have reached this goal, all of them presenting absence or instability for this phoneme, in line with evidence presented by Blamey et al. [56] of inconsistency in the acquisition of this phoneme several years after implantation. We also found a situation in which a primitive phoneme such as/t/ is completely absent in speech production.

Finally, the children with CIs had more absences of sounds than peers with NH. Whereas in the NH group a sound is absent in no more than 10% of children (the most challenging result /ʃ/, /ʦ/ and /ʎ/), this percentage is higher in the CI group, such as /ʎ/ being absent in 31.25%, and /z/ and /ʦ/ absent in 18.75% of the cases. These fragilities have been found previously in hearing aid users rather than in children with CIs [77]. For children with CIs, the most challenging modes of articulation concern fricative and affricate sounds and alveolar coarticulation place. These results align with Yang et al. [72].

We found a heterogeneous phonetic profile among the children with CIs. For example, one child had not acquired the alveolar phoneme /t/, which is one of the first typically produced since it is commonly present in babbling productions in typical development and, according to Iyer et al. [63], should be acquired three months after implantation in early implanted children. Interestingly, this child’s phonetic inventory differs from all the others since no acquired phonemes were found. This delay in phonetic development could be negatively influenced by later implantation (at 43 months) and/or could be partially caused by the presence of fragility in the linguistic area itself since, as suggested by Hardman et colleagues [8], language delays or disorders could co-occur with deafness.

In mastering the phonological system, children use phonological simplification processes, physiologically, during the development of their linguistic phonological system. The presence of these substitutions or omissions, however, can compromise speech intelligibility. As observed as mean scores, children with CIs use more phonological simplification processes than peers with NH, suggesting they are still building up their phonological system [80,81,82].

Our results on the prevalence of processes in the two groups—the children with CIs gaining equal or lower scores than their peers with NH—confirm the fragilities in phonological acquisition [65,67]. The higher percentage of the affrication process for children with CIs may be partly related to their acquiring fricative and affricate sounds later [56,67]. More children with CIs than their peers with NH presented substitution between frontal and posterior points of articulation, perhaps partially due to difficulties in perceiving the contrast between these sounds, in line with Johnson et al. [48]. A difference can also be found between the CI and the NH children in substitutions of couples of sounds differing for voicing/voiceless features such as d/t/ and /d/ or /p/ and /b/. The prevalence of sonorizations and desonorizations in the children with CIs was more than twice that in the children with NH, perhaps because of the difficulties experienced by children with CIs in perceiving these phoneme contrasts, as found by Eshaghi et al. [55]. Idiosyncratic processes seen in some of the CI children may negatively influence the child’s speech intelligibility, especially if other processes and/or incomplete inventory also occur, as found in other studies [98,99,100].

Regarding word structure processes, the children with CIs made significantly more syllable deletions than peers with NH, and all children with CIs made vowel or consonant omissions, compared with only 40% of peers with NH. It is interesting to note that, while the occurrence of consonant substitution is similar in both groups, vowel substitution is more frequent in children with CIs than in peers with NH. This result was unexpected since vowel identification is easier in Italian than consonant identification [88]. We had hypothesized for this reason that hearing loss would not negatively affect the skills of children with CIs in processing this type of sound. Reductions of two adjusted vocals and consonants are similar between the two groups. Indeed, almost a third of all children make diphthong reduction, while consonant cluster reduction seems to be one of the most common processes in both groups, being present in all children with CIs. Both NH and CI groups presented mostly substitutions and consonant cluster reduction, confirming that this is a prevalent process in kindergarten children’s speech [95], but while children with NH make almost 10% of frications among all processes, children with CIs present many consonant cancelations. This result is in line with the literature, confirming both the absence of a major difference between CI and NH children in consonant cluster production [96,97] and difficulties for children with CIs in coarticulating two adjusted consonants [94].

Considering how much a single phonetic process occurs in the children’s speech, the NH and CI groups both presented, on average, large numbers of consonant substitution and cluster reduction. However, while children with CIs also present many vowel or consonant omissions, their peers with NH present frication processes, while stopping is not one of the main processes displayed, in contrast with Flipsen and Parker [89]. Among the most common processes found in unintelligible children, the CI group displayed more consonant/vowel deletions; we can, therefore, hypothesize that this process negatively impacts their speech intelligibility, as stated by Hodson and Paden [101]. Overall, our results show more correct words and fewer simplified words in children with NH than in children with CIs.

Regarding emergent literacy, our results show significant differences between the two groups in phonological awareness tasks, in line with the literature [113,114,115]. Specifically, while there were significant differences in syllable segmentation, the differences in syllable blending did not reach a significant level, in contrast with Lee [122], probably due to the lower results of children with NH in this task, which is more complex than syllable segmentation [116] or due to the effect of verbal rehabilitation program participation as reported in the literature [102,103,104,105]. Regarding print knowledge, the groups showed similar scores both in syllable and vowel identification tasks, in line with previous research [115]. The similarity of the CI children’s scores in these tasks could be because the visual characteristics of the stimuli in these tasks provide assistance not available in phonological awareness tasks, which may involve phonological representations in verbal working memory.

These findings are supported by the results emerging through correlational analysis. In fact, associations were found in both groups between phonetic/phonological skills and emergent literacy, although some differences emerged. Specifically, phonetic/phonological skills are mostly associated with metaphonological skills in children with CIs, while in NH children they are associated with performance in print knowledge. As reported in the literature [140,141,142] and as discussed above for the similar performance in print knowledge tasks, children with CIs rely on different mechanisms to perform syllable and vowel identification tasks in cases of visual stimuli. These children could use an identification process that activates more visual memory, rather than a phonological one, and their emergent literacy could be boosted by non-auditory factors. Consequently, visual syllable and vowel identification could be completed without a grapheme-to-phoneme analysis of the input; in contrast, in print stimuli identification, children with NH could be more influenced by phonological mechanisms and memory.

Moreover, the phonological skills in children with CIs (number of unintelligible words, which indicate lower phonetic/phonological abilities) seem to negatively impact their scores in the phonological awareness task (which does not include visual cues), in line with Antia et al. [130]. This result confirms that speech impairments can make it more challenging to develop awareness of sounds in spoken language and the ability to work with them.

Overall, we found a relationship between emergent literacy and phonetic/phonological skills in both groups, but for children with CIs, other components, such as visual analysis of the word, can probably partially compensate for auditory difficulties in syllable recognition tasks. In children with NH, linguistic skills such as phonological knowledge usually help to develop emergent literacy skills. In children with CIs, this kind of support for written language identification ability seems less consistent.

Concerning the last aim of the study, the relationship between individual factors and the skills considered, earlier implantation seems to promote better phonetic and phonological development, as reported in other studies [21,22,23,24]. We also found moderate correlations between age at implantation and syllable identification, suggesting that hearing-impaired children receive help from oral language exposure in developing emergent literacy skills [115,116,130,138]. This finding, too, could be interpreted in terms of the different types of identification processes (visual analysis versus auditory analysis). Specifically, children with CIs who are implanted later might need to rely on a visual analysis strategy more than children who are implanted earlier, since the substantial lack of hearing experience can negatively impact phonological awareness.

This study has potential limitations. First, because of the exclusion criteria, the number of participants recruited is small. Second, it was impossible to recruit a third group of participants, who would be matched with the CI group for hearing age instead of chronological age. Third, we did not collect direct measures of the children’s visual analysis of the stimuli, since it was not the aim of this study. Finally, we did not collect non-verbal IQ data for the participants, and we cannot exclude the possibility that differences in cognitive abilities may have influenced the outcomes. Future research should address these limitations with larger groups of participants.

## 5. Conclusions

In summary, the present study describes both quantitatively and qualitatively the phonetic/phonological skills and emergent literacy development in Italian-speaking children with CIs, helping to close the gap in the literature on this topic. This study can be relevant both for literacy and rehabilitation clinicians. It will help them develop appropriate language evaluation and intervention programs, considering the similarities and differences between children with NH and children with CIs and the role of other skills that can compensate for auditory difficulties in mastering early literacy skills. It will also raise awareness among the relevant professionals of the most critical phoneme acquisition and phonological processes commonly occurring in the speech of children with CIs. This will assist in planning and implementing rehabilitation strategies to reduce limitations in intelligibility, thus helping the children acquire better communication skills. Given the influence of phonetic/phonological difficulties on later literacy outcomes, a specific and targeted intervention can help prevent future literacy difficulties.

## Figures and Tables

**Table 1:** Studies reporting the phonetic inventory composition of CI users and NH peers (although variable, benchmark criterion adopted in the studies to consider a consonant mastered was at least 70% of the participants).

	Hearing Age (Months)	Language	CI Group	NH Peers
Iyer et al., 2016 [63]	24	English	/b/; /m/; /d/; /w/; /p/; /t/; /k/; /n/; /j/; /g/	/b/; /m/; /d/; /w/; /t/; /k/; /n/; /g/; /s/; /l/
Sundarrajan et al., 2019 [23]	24	English	/b/; /p/; /m/; /n/; /j/; /w/; /k/; /g/; /t/; /d/; /h/; /s; /f/; /r/; /l/	/b/; /p/; /m/; /n/; /j/; /w/; /k/; /g/; /t/; /d/; /h/; /s/; /f/; /r/; /l/; /ʃ/; /z/; /v/; /ʧ/; /ð/
Serry et Blamey, 1999 [58] (for NH peers: McLeod & Crowe, 2018 [64])	48	English	/m/; /j/; /w/; /b/; /n/; /d/; /p/; /l/; /ʃ/; /h/; /θ/; /ʒ/	/b/; /p/; /m/; /n/; /j/; /w/; /k/; /g/; /t/; /d/; /h/; /s/; /f/; /r/; /l/; /ʃ/; /z/; /v/; /ʧ/; /ŋ/; /ʤ/; /ʒ/; /ɹ/
Spencer & Guo, 2012 [65] (for NH peers: Smit et al., 1990 [66])	48	English	/p/; /b/; /t/; /d/; /k/; /g/; /m/; /n/; /w/; /f/; /s/; /ʃ/; /h/; /tʃ/	/p/; /b/; /t/; /d/; /k/; /g/; /m/; /n/; /w/; /j/; /f/; /v/; /s/; /ʃ/; /h/; /dʒ/
Lynce et al., 2019 [67] (for NH peers: Silva et al., 2012 [69])	48	Portuguese	/p/; /k/; /f/; /ʃ/; /ʃ/; /b/; /d/; /m/; /l/; /r/; /ɲ/	/p/; /b/; /t/; /d/; /k/; /g/; /m/; /n/; /ʧ/; /ʤ/; /f/; /v/; /s/; /z/; /ʃ/; /ʒ/; /ʎ/; /ɲ/; /ʀ/; /ɾ/; /l/
Bouchard et al., 2007 [68] (for NH peers: Vinter, 2001 [71])	18	French	/m/;/p/;/l/;/b/;/n/;/w/;/t/;/j/;/d/;/s/;/r/;/s/	/p/;/b/;/t/;/d/;/k/;/m/;/n/;/l/;/s/;/w/;/j/;/r/
Sohrabi & Jalilevand, 2021 [59]	62 *	Persian	/p/; /b/; /t/; /d/; /ʔ/; /m/; /n/; /h/	/p/; /b/; /t/; /d/; /c/; /g/; /q/; /ʔ/; /m/; /n/; /s/; /z/; /ʃ/; /f/; /v/; /x/; /h/; /ʧ/; /ʤ/; /l/; /j/; /r/
Lee et al., 2022 [10] (for NH peers: Cho, 2008 [70])	24	Korean	/g/; /k^=^/; /n/; /d/; /t^=^/; /ɾ/; /m/; /b/; /p^=^/; /s/; /ʥ/; /ʨ^=^/; /ʨ^h^/; /k^h^/; /t^h^/; /p^h^/; /h/; /k ̚/; /n/; /t ̚/; /l/; /p ̚/; /ŋ/	/k ̚/; /n/; /t ̚/; /l/; /m/; /p ̚/; /ŋ/; /g/; /k^=^/; /n/; /d/; /t^=^/; /ɾ/; /m/; /b/; /p^=^/; /s/; /ʥ/; /ʨ^=^/; /ʨ^h^/; /k^h^/; /t^h^/; /p^h^/; /h/

Note. * Chronological age.

**Table 2:** Descriptive statistics split by the two groups and results of nonparametric *t*-test (Mann–Whitney U) for independent samples at the PFLI and early literacy test.

		Group			
	CI	NH	*U*	*p*	Effect Size (Cohen’s *d*)
	*M* (*SD*)	*M* (*SD*)			
Simplified Words	84.56 (51.74)	38.65 (45.20)	67.0	**0.003**	0.58
Unintelligible Words	5.06 (6.29)	0.65 (1.27)	80.0	**0.005**	0.50
Phonemes (acquired)	15.59 (5.02)	18.65 (2.06)	85.0	**0.016**	0.47
CMF Syllable Segmentation	7.63 (6.69)	13.45 (1.36)	84.5	**0.015**	0.47
CMF Syllable Blending	7.63 (6.62)	10.95 (5.12)	121.0	0.214	0.28
Syllable Identification	5.31 (5.70)	5.80 (7.67)	145.0	0.631	0.11
Vowel Identification	2.75 (1.98)	3.55 (1.73)	120.5	0.200	0.25

Note. *N* = 36. CIs = Children with cochlear implants. NH = Children with normal hearing. Significant values (*p* < 0.05) are in bold.

**Table 3:** Spearman’s Correlations for the CI Group between Phonological Skills and Early Literacy Skills.

	1	2	3	4	5	6	7
1. PFLI Simplified Words	-	0.73 **	0.39	−0.46	−0.28	0.25	−0.03
2. PFLI Unintelligible Words		-	−0.011	−0.58 *	−0.55 *	0.20	−0.08
3. PFLI Phonemes (acquired)			-	0.17	0.35	0.006	0.10
4. CMF Syllable Segmentation				-	0.92 **	−0.11	0.51 *
5. CMF Syllable Blending					-	−0.10	0.45
6. Syllable Identification						-	0.46
7. Vowel Identification							-

Note. *N* = 16. Significant values (*p* < 0.05) are in bold * p < 0.05, ** *p* < 0.01.

**Table 4:** Spearman’s Correlations for the NH Group between Phonological Skills and Early Literacy Skills.

	1	2	3	4	5	6	7
1. PFLI Simplified Words	-	−0.002	−0.33	−0.09	−0.026	−0.38	−0.48 *
2. PFLI Unintelligible Words		-	−0.05	0.10	−0.21	−0.44	−0.21
3. PFLI Phonemes (acquired)			-	0.23	0.25	0.45 *	0.50 *
4. CMF Syllable Segmentation				-	0.61 **	−0.37	−0.03
5. CMF Syllable Blending					-	0.03	0.31
6. Syllable Identification						-	0.61 **
7. Vowel Identification							-

Note. *N* = 20. Significant values (*p* < 0.05) are in bold * p < 0.05, ** *p* < 0.01.

## Data Availability

The dataset used in this study is available on request.

## Abbreviations

The following abbreviations are used in this manuscript:

CI	Cochlear Implant
NH	Normal Hearing
DHH	Deaf and Hard of Hearding
CMF	Valutazione delle Competenze Metafonologiche
PFLI	Prove per la Valutazione Fonologica del Linguaggio Infantile

## Appendix A

**Table A1:** Phonetic inventory in the CI group and the NH group.

			CI Group						NH Group					
			Stable		Emergent		Unstable		Stable		Emergent		Unstable	
			(Raw)	%	(Raw)	%	(Raw)	%	(Raw)	%	(Raw)	%	(Raw)	%
Plosives	bilabial	/p/	(15)	93.7	(1)	6.2	(0)	0	(20)	100	(0)	0	(0)	0
		/b/	(15)	93.7	(1)	6.2	(0)	0	(20)	100	(0)	0	(0)	0
	dental	/t/	(15)	93.7	(0)	0	(1)	6.2	(20)	100	(0)	0	(0)	0
		/d/	(15)	93.7	(1)	6.2	(0)	0	(20)	100	(0)	0	(0)	0
	velar	/k/	(15)	93.7	(1)	6.2	(0)	0	(20)	100	(0)	0	(0)	0
		/g/	(10)	62.5	(5)	31.2	(1)	6.2	(20)	100	(0)	0	(0)	0
Fricatives	labio-dental	/f/	(15)	93.7	(1)	6.2	(0)	0	(19)	95	(1)	5	(0)	0
		/v/	(13)	81.2	(3)	18.7	(0)	0	(20)	100	(0)	0	(0)	0
	alveolar	/s/	(15)	93.7	(1)	6.2	(0)	0	(19)	95	(1)	5	(0)	0
		/z/	(0)	0	(13)	81.2	(3)	18.7	(5)	25	(15)	75	(0)	0
	palato-alveolar	/ʃ/	(15)	93.7	(1)	6.2	(0)	0	(14)	70	(4)	20	(2)	10
Affricatives	alveolar	/ts/	(6)	37.5	(7)	43.7	(3)	18.7	(9)	45	(9)	45	(2)	10
		/dz/	(15)	93.7	(1)	6.2	(0)	0	(3)	15	(16)	80	(1)	5
	palato-alveolar	/ʧ/	(13)	81.2	(2)	12.5	(1)	6.2	(18)	90	(1)	5	(1)	5
		/ʤ/	(15)	93.7	(1)	6.2	(0)	0	(18)	90	(1)	5	(1)	5
Nasals	bilabial	/m/	(15)	93.7	(1)	6.2	(0)	0	(20)	100	(0)	0	(0)	0
	alveolar	/n/	(15)	93.7	(1)	6.2	(0)	0	(20)	100	(0)	0	(0)	0
	palatal	/ɲ/	(10)	62.5	(4)	25	(2)	12.5	(18)	90	(2)	10	(0)	0
Laterals	alveolar	/l/	(15)	93.7	(1)	6.2	(0)	0	(20)	100	(0)	0	(0)	0
	palatal	/ʎ/	(2)	12.5	(9)	56.2	(5)	31.2	(1)	5	(17)	85	(2)	10
Trills	alveolari	/r/	(15)	93.7	(1)	6.2	(0)	0	(16)	80	(4)	20	(0)	0
Semi-consonants	palatal	/j/	(13)	81.2	(3)	18.7	(0)	0	(18)	90	(2)	10	(0)	0
	velar	/w/	(9)	56.2	(7)	43.7	(0)	0	(15)	75	(5)	25	(0)	0

**Table A2:** Simplification processes over total processes in the CI group and in the NH group.

	CI Group		NH Group	
	(raw)	%	(raw)	%
stopping	(4.29)	5.43%	(1.55)	2.78%
affrication	(2.12)	2.59%	(1.09)	3.43%
deaffrication	(1.65)	2.03%	(3.23)	10.14%
gliding	(1.65)	1.56%	(1.64)	6.06%
fronting	(1.24)	1.55%	(0.95)	1.65%
palatalization	(0)	0.00%	(0.09)	0.07%
backing	(0.29)	0.31%	(0.09)	0.58%
voicing	(0.76)	1.14%	(0.23)	0.83%
devoicing	(1.94)	1.66%	(0.32)	1.91%
weak syllable delection	(3.12)	4.19%	(0.41)	0.42%
cons./vow. omission	(11.12)	15.72%	(3.36)	7.87%
metathesis	(0.41)	0.51%	(0.14)	0.76%
epenthesis	(1.12)	2.27%	(0.36)	0.95%
diphthong reduction	(0.29)	0.77%	(0.50)	3.62%
consonant harmony	(2.76)	4.16%	(1.00)	2.95%
consonant substitution	(11.47)	20.37%	(8.09)	24.08%
vowel substitution	(2.12)	2.61%	(0.59)	1.30%
cluster reduction	(15.71)	21.06%	(14.18)	25.33%
atypical processes	(0.35)	0.24%	(0.18)	0.53%
idyosincratic processes	(4.88)	5.19%	(0.82)	1.34%

Note. *N*_CI_ = 16. *N*_NH_ = 20.

**Table A3:** Children producing simplification processes in the CI group and in the NH group.

	CI Group		NH Group	
	(Raw Mean)	%	(Raw Mean)	%
stopping	(9)	56.25%	(7)	35%
affrication	(9)	56.25%	(5)	25%
deaffrication	(9)	56.25%	(11)	55%
gliding	(7)	43.75%	(10)	50%
fronting	(6)	37.50%	(4)	20%
palatalization	(0)	0%	(1)	5%
backing	(4)	25%	(1)	5%
voicing	(7)	43.75%	(3)	15%
devoicing	(7)	43.75%	(5)	25%
weak syllable delection	(11)	68.75%	(3)	15%
cons./vow. omission	(15)	93.75%	(12)	60%
metathesis	(5)	31.25%	(3)	15%
epenthesis	(12)	75%	(4)	20%
diphthong reduction	(5)	31.25%	(6)	30%
consonant harmony	(10)	62.50%	(8)	40%
consonant substitution	(14)	87.50%	(19)	95%
vowel substitution	(11)	68.75%	(7)	35%
cluster reduction	(15)	93.75%	(17)	85%
atypical processes	(3)	18.75%	(2)	10%
idyosincratic processes	(8)	50%	(7)	35%

Note. *N*_CI_ = 16. *N*_NH_ = 20.
